# Monitoring Leaf Nitrogen Accumulation With Optimized Spectral Index in Winter Wheat Under Different Irrigation Regimes

**DOI:** 10.3389/fpls.2022.913240

**Published:** 2022-06-17

**Authors:** Hui Sun, Meichen Feng, Wude Yang, Rutian Bi, Jingjing Sun, Chunqi Zhao, Lujie Xiao, Chao Wang, Muhammad Saleem Kubar

**Affiliations:** ^1^College of Resource and Environment, Shanxi Agricultural University, Jinzhong, China; ^2^College of Agriculture, Shanxi Agricultural University, Jinzhong, China; ^3^College of Arts and Science, Shanxi Agricultural University, Jinzhong, China

**Keywords:** leaf nitrogen accumulation, optimized spectral index, band combination, spectral transformation method, winter wheat

## Abstract

Rapid and non-destructive estimation of leaf nitrogen accumulation (LNA) is essential to field nitrogen management. Currently, many vegetation indices have been used for indicating nitrogen status. Few studies systematically analyzed the performance of vegetation indices of winter wheat in estimating LNA under different irrigation regimes. This study aimed to develop a new spectral index for LNA estimation. In this study, 2 years of field experiments with different irrigation regimes were conducted from 2015 to 2017. The original reflectance (OR) and three transformed spectra [e.g., the first derivative reflectance (FDR), logarithm of the reciprocal of the spectra (Log(1/*R*)), and continuum removal (CR)] were used to calculate two- and three-band spectral indices. Correlation analyses and univariate linear and non-linear regression between transformed-based spectral indices and LNA were performed. The performance of the optimal spectral index was evaluated with classical vegetation index. The results showed that FDR was the most stable transformation method, which can effectively enhance the relationships to LNA and improve prediction performance. With a linear relationship with LNA, FDR-based three-band spectral index 1 (FDR-TBI1) (451, 706, 688) generated the best performance with coefficient of determination (*R*^2^) of 0.73 and 0.79, the root mean square error (RMSE) of 1.267 and 1.266 g/m^2^, and the ratio of performance to interquartile distance (RPIQ) of 2.84 and 2.71 in calibration and validation datasets, respectively. The optimized spectral index [FDR-TBI1 (451, 706, 688)] is more effective and might be recommended as an indicator for estimating winter wheat LNA under different irrigation regimes.

## Introduction

Nitrogen is one of the essential nutrients for the crop growth and development, determining the yield, and grain quality. At present, excessive nitrogen application has adverse effects on the environment and may result in the low nitrogen use efficiency. Leaf nitrogen accumulation (LNA), the comprehensive information of nitrogen content and dry matter, effectively reflects the crop population nitrogen information. Timely and accurate estimation of LNA is significant for nitrogen nutrition diagnosis, field decision-making, and improvement of grain yield, and quality in winter wheat (Zhao et al., 2005).

Traditional method of nitrogen content analysis, through chemical method in laboratory, is time-consuming and laborious. The application of optical techniques provided a rapid and non-destructive way for *in situ* nitrogen diagnosis. In term of leaves, nitrogen status of individual plant could be inferred from the parameters measured by SPAD-502 or Dualex-3 (Esfahani et al., 2008; Tremblay et al., 2012). However, to avoid poor representation of one or two leaves, a large number of sample replications were required to be measured to extract the robust information. In contrast, remote sensing technology, as an important technology of precision agriculture, can rapidly assess the field crop population phenotypic information in large area by capturing canopy information. It has been applied to the growth monitoring (Dong et al., 2019), nutrition diagnosis (Liang et al., 2018), and yield prediction (Zhang et al., 2019).

Vegetation index is an important tool for the crop phenotype monitoring with ground-based and satellite remote sensing because of its universality and efficient computation (Tian et al., 2011). Canopy reflectance can be used in the estimation of nitrogen status on the basic of the close relationship between nitrogen and chlorophyll content at canopy level. Due to the strong absorption in the red region and high reflectance in the near-infrared region of green plant, the red and near-infrared bands were commonly used for spectral index (e.g., NDVI, RVI) for plant growth monitoring. Zhu et al. (2008) reported that RVI (870, 660) and RVI (810, 660) had high correlation with LNA in both rice and wheat. Because of the high correlation between blue, green, and red-edge region with plant nitrogen status, some two-band vegetation indices were applied to nitrogen monitoring, e.g., green normalized difference vegetation index (GNDVI) (Bronson et al., 2003), normalized difference red-edge index (NDRE) (Thompson et al., 2015), red-edge chlorophyll index (CI_rededge_) (Clevers and Kooistra, 2012), ratio index (RI-1dB) (He et al., 2016), and normalized pigment chlorophyll index (NPCI) (Xu et al., 2014). Reyniers et al. (2006) constructed an optimized vegetation index (VI_opt_) to predict wheat nitrogen with a multispectral radiometer.

Except for the two-band spectral indices, there had three-band spectral indices (TBIs) proposed for nitrogen estimation. R_705_/(R_717_+R_491_) developed by Tian et al. (2011) is a good indicator of rice leaf nitrogen content at ground and space level. Chen et al. (2010) developed the double-peak canopy nitrogen index (DNCI) to better assess nitrogen efficiency in maize and wheat by minimizing the LAI influence. It is reported that three-band vegetation index could reduce the saturation effect of two-band vegetation index and increase sensitivity and prediction accuracy. Wang et al. (2012) proposed (R_924_-R_703_+2^*^R_423_)/(R_924_+R_703_+2^*^R_423_) to decrease the saturation of two-band spectral index, increasing stability and accuracy of leaf nitrogen content prediction. Schlemmer et al. (2013) found that medium resolution imaging spectrometer terrestrial chlorophyll index (MTCI) had higher accuracy than NDVI and EVI in estimating maize canopy nitrogen content. However, when the canopy nitrogen content is above 6 g/m^2^, CI_green_ was recommended. Zheng et al. (2018) found that DATT and CI_rededge_ had consistent good performance in estimating LNA. The relationship between DATT and LNA was non-linear, whereas CI_rededge_ was linear, respectively. Therefore, in the aspect of the advantages of the three-band vegetation index, further study and verification were necessary.

As an important step in spectral data analysis, spectral transformation techniques have been used to enhance spectral characters and reduce the influence of interference factors. For instance, Clark and Roush (1984) pointed out that those absorption features of not interest can be removed by continuum-removal (CR) analysis, thereby isolating individual absorption features. The logarithm of the reciprocal of the spectra (Log(1/*R*)) can highlight spectral differences in the visible region, minimizing the influence of illumination variation (Wang et al., 2009). First derivative reflectance (FDR) method is effective not only in removing the influence of background but also in resolving the overlapping signals and enhancing subtle peaks (Al-Moustafa et al., 2012; Liaghat et al., 2014; Meng et al., 2020). Previous studies had introduced spectral transformation into the construction of spectral index for higher and stable prediction. Wen et al. (2019) indicated that two-band spectral indices using FDR performed better in estimating leaf nitrogen content of maize across four growth stages. In addition, based on Log(1/*R*), NDNI ([log(1/*R*_1510_) – log(1/*R*_1680_)]/[log(1/*R*_1510_) + log(1/*R*_1680_)]) were proposed to predict canopy nitrogen, especially in low vegetation continuous canopies (Serrano et al., 2002). Li D. et al. (2016) founded that the optimized CR-based vegetation index can be used to monitor the leaf nitrogen content of litchi. Therefore, the combination of spectral transformation and spectral index can synthesize their advantages and is a potential way to construct new spectral indices. However, few studies have systematically investigated the effects of multiple spectral transformation methods on the construction of spectral index.

To date, many vegetation indices have been used to indicate nitrogen status. Nevertheless, the efficiency of the vegetation indices was affected by season, growth, and cultivation environmental condition (Liaghat et al., 2014). Irrigation and nitrogen fertilizers are the main controlling factors in crop production. Few studies systematically analyzed the performance of vegetation indices of winter wheat in estimating LNA under different irrigation regimes. For achieving this, winter wheat under five irrigation regimes were set as the object for nitrogen status estimation. This study aimed to (1) study the relationships between LNA and spectrum with or without the transformations; (2) develop new spectral indices calculated from the spectrum with or without transformations for LNA estimation; and (3) obtain the optimal spectral index and model for LNA estimation of winter wheat for different irrigation regimes.

## Materials and Methods

### Site Description and Experimental Design

The experiment was conducted from 2015 to 2017 at the experiment station of Shanxi Agricultural University (E112°33′, N37°25′), located at Taigu county of Shanxi Province, China. The experiment site has a temperate continental climate with an average annual temperature of 9.8°C, 175 frost-free days and annual precipitation about 450 mm. The experiment was carried out in a bottomless pool made of waterproof cement. There had a steel frame rain-proof shelter above the experiment pool. The refilled soil is classified as calcareous cinnamon soil (Alfisols in US taxonomy) with organic matter content of 9.60 g kg^−1^. The mean available nitrogen, phosphate, and potassium contents were 57.75, 22.10, and 185.48 mg kg^−1^, respectively. In the artificial root zone, the field capacity of and the bulk density were 24.24% and 1.42 g cm^−3^, respectively.

**Figure 1:**
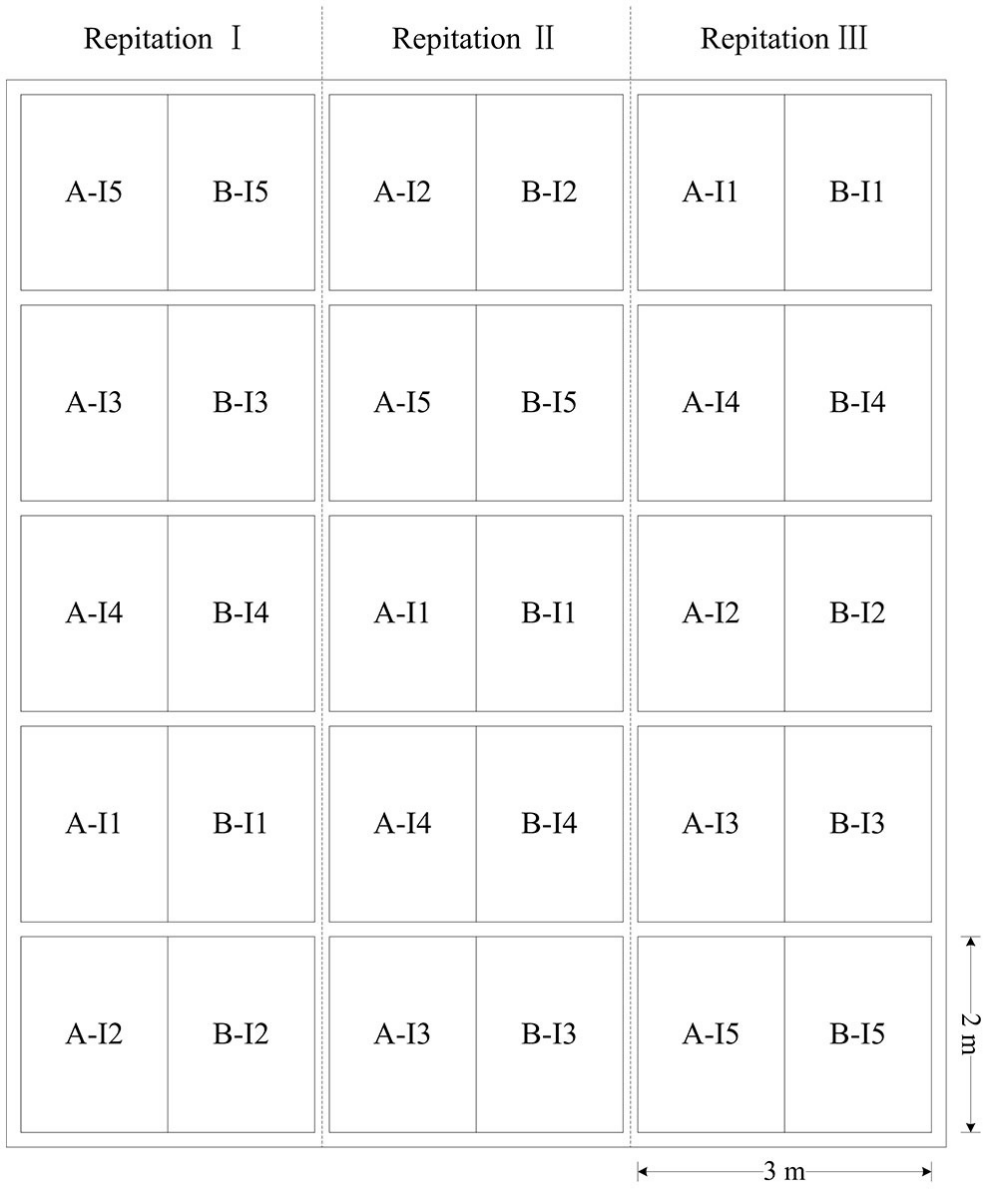
The distribution map of the different treatments of irrigation regimes applied in the study. I_1_ represents four irrigations at jointing, booting, flowering, and filling stage. I_2_ represents three irrigations at jointing, booting, and filling stage. I_3_ represents two irrigations at jointing and flowering stage. I_4_ represents two irrigations at jointing and filling stage. I_5_ represents the treatment without irrigation. In 2015–2016, A and B represent Chang 4738 and Zhongmai 175, whereas in 2016–2017 both A and B represent Jingdong 17.

The experiment was set up in a randomized complete block design with three replications. Five irrigation regimes were applied in the 2-year experiment: I_1_ (four irrigations at jointing stage, booting stage, flowering stage, and filling stage), I_2_ (three irrigations at jointing stage, booting stage, and filling stage), I_3_ (two irrigations at jointing stage and flowering stage), I_4_ (two irrigations at jointing stage and booting stage), and I_5_ (without irrigation). The upper limit of each irrigation was 80% of the soil field capacity, and the water consumption was controlled with a water meter. The growth stages for irrigation were selected based on the study of Zadoks et al. (1974). Two winter wheat cultivars (Chang 4738 and Zhongmai 175) were sown in rows spaced 20 cm on September 29, 2015. And only one cultivar (Jingdong 17) was sown on October 1, 2016. Each plot was 2 m wide and 3 m long in 2015 and 1.5 m wide and 2 m long in 2016 (Figure 1). For all treatments, the fertilizers were applied prior to seeding with 150 kg N hm^−2^, 150 kg P_2_O_5_ hm^−2^, and 150 kg K_2_O hm^−2^. Field managements were consistent with the local standard practice for winter wheat.

### Canopy Reflectance Measurement

The canopy reflectance was collected during the major growth stages (from jointing to filling stage) by a FieldSpec 3.0 spectrometer [Analytical Spectral Devices (ASD), Boulder, CO, USA] at 1 m above the canopy of winter wheat. The instrument field angle is 25° over the wavelength of 350–2,500 nm, with a sampling interval of 1.4 nm and spectral resolution of 3 nm between 350 and 1,000 nm; and a sampling interval of 2 nm and spectral resolution of 10 nm between 1,000 and 2,500 nm. The measurements were taken under clear sky conditions from 10:00 to 14:00. A 40 × 40 cm BaSO_4_ panel was used for calibrating the baseline reflectance before each measurement. Three measurements were taken in each plot, with 10 scans for each measurement. All reflectance curves within a plot were averaged to represent the spectrum in each plot.

### Leaf Nitrogen Accumulation Measurement

After the spectra measurements, plant samples in 20 cm lengths were collected from the soil surface and stored in plastic bags. In the laboratory, all leaves were picked and dried in an oven at 105°C for half an hour, then at 80°C for 24 h to the constant weight and reweighed to get the leaf's dry weight. The dried leaves were dissolved by concentrated sulfuric acid, and the leaf nitrogen concentration was measured by intermittent automatic chemistry analyzer (SmartChem 200, AMS, Italy). The LNA was calculated by the formula,


(1) $$\begin{array}{r} \text{LNA}=\text{LNC}\;\times \;\text{DW} \end{array}$$

where LNC is the leaf nitrogen concentration, and DW is dry weight of leaves per unit ground area.

The normality of the distribution of LNA data was test by using the Kolmogorov–Smirnov (K–S) test in SPSS 19.0 (SPSS Inc., Chicago, United States).

### Data Pretreatment and Calculation of Spectral Indices

In order to reduce the instrument noise and interference effect of atmosphere, the bands in 350–400, 1,350–1,400, 1,800–1,950, and 2,450–2,500 nm were removed for the subsequent spectral analysis. Three frequently used transformation methods (the first derivative (FD), logarithm of reciprocal (Log(1/*R*)), and CR) were implemented on the original reflectance (OR) to eliminate other component noise or enhance the absorption characteristics of the target (Li L. et al., 2016). The CR transformation was implemented with ENVI 4.2 software.

Most of the existing vegetation indices were developed from three vegetation indices (normalized differences vegetation index, difference vegetation index, and ratio vegetation index). Vegetation indices in the form of CI were commonly used to estimate nitrogen status. By adding a constant, soil-adjusted vegetation index reduced the effect of soil background (Rondeaux et al., 1996), whereas modified CI (MCI) improved prediction accuracy in both low and high coverage (Zhang et al., 2021). Therefore, six two-band forms of spectral indices [normalized difference spectral index (NDSI), difference spectral index (DSI), ratio spectral index (RSI), chlorophyll spectral index (CSI), soil-adjusted spectral index (SASI), and modified chlorophyll spectral index (MCSI)] were selected for optimization of bands. Furthermore, due to the advantages of the three-band vegetation index mentioned above, five forms of TBIs were selected. The formulae of these spectral indices were listed in Table 1. The random band combination method is an effective way to improve the performance of classical vegetation indices (Yu et al., 2013; Hasituya et al., 2020). In this study, based on OR, FDR, Log(1/*R*), and CR, two-band forms of spectral indices were calculated with all possible combinations of two bands in 400–2,450 nm (excluding 1,350–1,400, and 1,800–1,950 nm) at 1-nm interval. For the high correlation between adjacent bands, one out of three bands was reserved for screening optimal three-band combination spectral indices. The relationships between spectral indices and LNA were calculated in MATLAB R2010b and depicted in contour maps. In this study, 14 classical vegetation indices for nitrogen status estimation (Table 2) were chosen to verify the predictive ability of the optimized spectral index.

**Table 1:** The formulae of selected two-band and three-band spectral index in this study.

**Categories**	**Spectral** **index**	**Formula**
Two-band spectral index	NDSI	NDSI = (*R*_*i*_−*R*_*j*_)/(*R*_*i*_+*R*_*j*_)
	DSI	DSI = *R*_*i*_−*R*_*j*_
	RSI	RSI = *R*_*i*_/*R*_*j*_
	SASI	SASI = (1+*L*) × (*R*_*i*_−*Rj*)/(*R*_*i*_+*R*_*j*_+*L*)*Lϵ*(0, 1)
	CSI	CSI = (*R*_*i*_−*R*_*j*_)/*R*_*j*_
	MCSI	MCSI = (*R*_*i*_−*R*_*j*_)/(*R*_*j*_+*M*)*M*∈(−3, 6)
Three-band spectral index	TBI1	TBI1 = (*R*_*i*_−*R*_*j*_)/(*R*_*i*_+*R*_*k*_)
	TBI2	TBI2 = *R*_*i*_/(*R*_*j*_+*R*_*k*_)
	TBI3	TBI3 = *R*_*i*_/(*R*_*j*_ × *R*_*k*_)
	TBI4	TBI4 = (*R*_*i*_−*R*_*j*_)/(*R*_*j*_−*R*_*k*_)
	TBI5	TBI5 = (*R*_*i*_−*R*_*j*_)/(*R*_*i*_+*R*_*j*_−2 × *R*_*k*_)

*R_i_, R_j_, and R_k_ represent the reflectance value with no or different transformation methods at i, j, and k nm, respectively*.

**Table 2:** Classical vegetation indices used in this study.

**Vegetation index**	**Formula**	**References**
Double-peak canopy nitrogen index (DCNI)	(*R*_720_−*R*_700_)/(*R*_700_−*R*_670_)/(*R*_720_−*R*_670_+0.03)	Chen et al., 2010
Green normalized difference vegetation index (GNDVI)	(*R*_750_−*R*_550_)/(*R*_750_+*R*_550_)	Gitelson and Merzlyak, 1998
Green chlorophyll index (CI_green_)	*R*_790_/*R*_550_−1	Gitelson et al., 2005
Red edge chlorophyll index (CI_rededge_)	*R*_790_/*R*_720_−1	Gitelson et al., 2005
Enhanced vegetation index (EVI2)	2.5 × (*R*_800_−*R*_660_)/(1+*R*_800_+2.4 × *R*_660_)	Jiang et al., 2008
R810/R660	*R*_810_/*R*_660_	Zhu et al., 2008
RI_ldB	*R*_735_/*R*_720_	He et al., 2016
MCARI2/OSAVI2	[(*R*_750_−*R*_705_)−0.2 × (*R*_750_−*R*_705_)] × (*R*_750_/*R*_705_)/[(1+0.16) × (*R*_750_−*R*_705_)/(*R*_750_+*R*_705_+0.16)]	Wu et al., 2008
Optimal soil-adjusted vegetation index (OSAVI)	(1+*L*) × (*R*_800_−*R*_670_)/(*R*_800_+*R*_670_+*L*)(*L* = 0.16)	Rondeaux et al., 1996
Normalized difference red-edge index (NDRE)	(*R*_790_−*R*_720_)/(*R*_790_+*R*_720_)	Fitzgerald et al., 2006
Water resistance N index (WRNI)	(*R*_735_−*R*_720_)/(*R*_735_+*R*_720_)/*FWBI*	Feng et al., 2016
Optimized vegetation index (VIopt)	$\left(1+0.45\right)\times \left({R_{800}}^{2}+1\right)/\left(R_{670}+0.45\right)$	Reyniers et al., 2006
Normalized pigment chlorophyll index (NPCI)	(*R*_430_−*R*_680_)/(*R*_430_+*R*_680_)	Peñuelas et al., 1994
(R_924_-R_703_+2*R_423_)/ (R_924_+R_703_+2*R_423_)	(*R*_924_−*R*_703_+2 × *R*_423_)/(*R*_924_+*R*_703_+2 × *R*_423_)	Wang et al., 2012

*The formula of FWBI (floating-position water band index) was R_900_/R_min(930−980)_*.

### Model Calibration and Validation

Data of winter wheat of two growing seasons from 2015 to 2017 were pooled in this study. In order to screen the optimal spectral index and construct the best estimating model of LNA, the dataset was randomly divided into two subsets: two-thirds for model calibration and one-third for model validation. Based on the calibration dataset, the quantitative relationship between spectral indices with optimal band combination and LNA was established by using the univariate linear and non-linear (logarithmic, parabolic, power, and exponential) regression models. And three metrics [coefficient of determination (*R*^2^), the root mean square error (RMSE), and the ratio of performance to interquartile distance (RPIQ) (Bellon-Maurel et al., 2010)] were calculated with the validation dataset to evaluate the accuracy and stability of the estimation model. In addition, the noise equivalent (NE) was calculated to further compare different vegetation indices. The formulae of these statistics were as follows,


(2) $$\begin{array}{ccc} R^{2} & = & \frac{\sum_{i=1}^{n}{\left(y_{ipre}-\bar{y}\right)}^{2}}{\sum_{i=1}^{n}{\left(y_{imea}-\bar{y}\right)}^{2}} \end{array}$$

(3) $$\begin{array}{ccc} RMSE & = & \sqrt{\frac{\sum_{i=1}^{n}{\left(y_{imea}-y_{ipre}\right)}^{2}}{n}} \end{array}$$

(4) $$\begin{array}{rcl} RPIQ & = & \frac{\text{Q}3-\text{Q}1}{\text{RMSE}} \end{array}$$

(5) $$\begin{array}{rcl} \text{NE}\Delta \text{LNA} & = & \frac{\text{RMSE}\left(\text{VI}vs.\;\text{LNA}\right)}{d\left(\text{VI}\right)/d\left(\text{LNA}\right)} \end{array}$$

where *y*_*imea*_, *y*_*ipre*_, and $\bar{y}$ are the measured LNA, predicted LNA values, and the average value of measured LNA, respectively; *n* is the number of samples; Q1 and Q3 are the first quartile and third quartile of the dataset, respectively; RMSE (VI vs. LNA) is the RMSE of the best-fit regression function of VI vs. LNA; *d*(VI)/*d*(LNA) is the FD of the established relationship; and the NE provides the dynamic changes of sensitivity of different spectral indices to LNA over the whole range (Viña and Gitelson, 2005).

## Results

### Response of LNA and Canopy Reflectance to Different Irrigation Regimes

**Figure 2:**
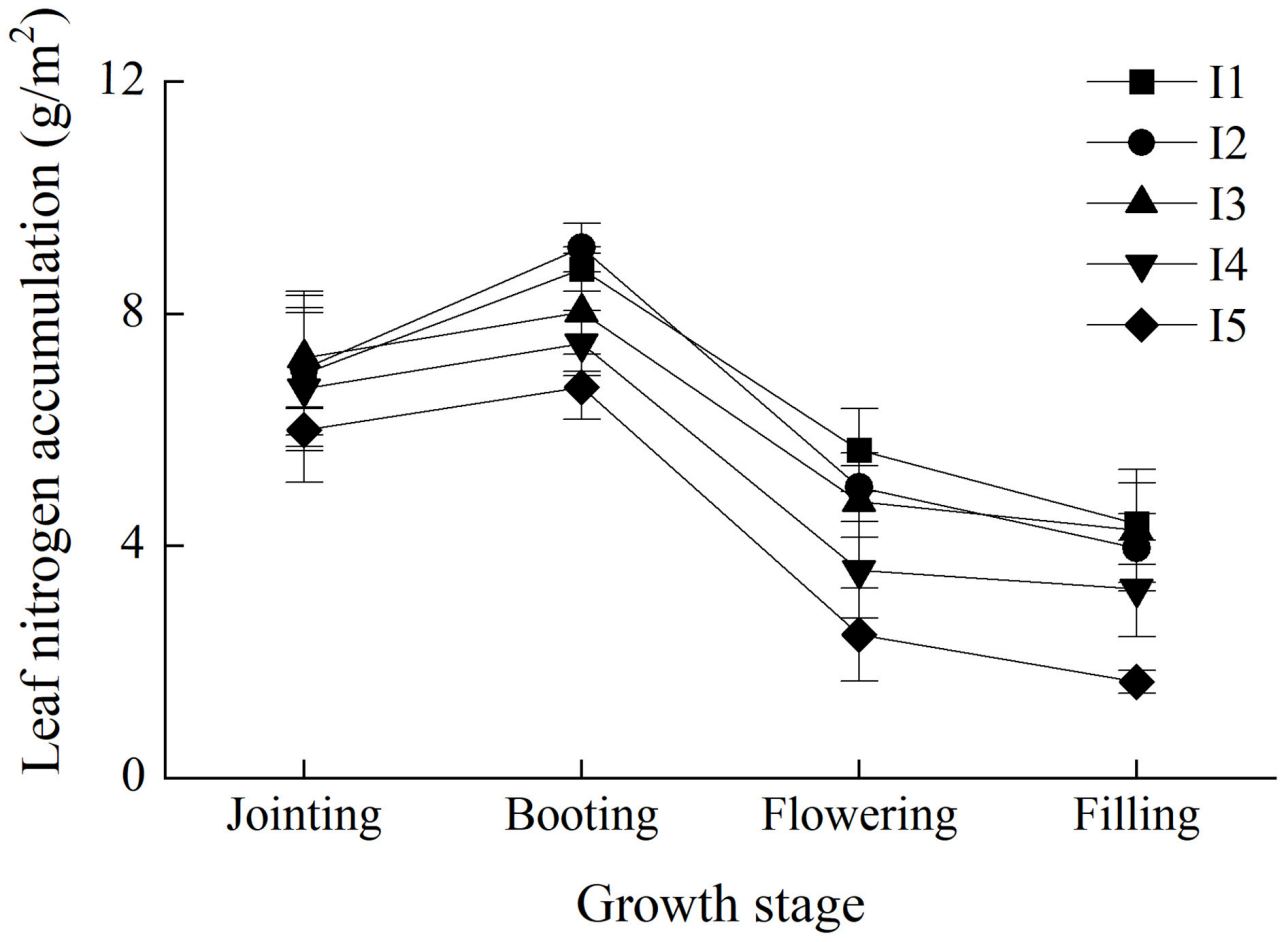
Change trends of the leaf nitrogen accumulation over growth progress under different experimental treatments in winter wheat. I_1_ represents four irrigations at jointing, booting, flowering, and filling stage. I_2_ represents three irrigations at jointing, booting, and filling stage. I_3_ represents two irrigations at jointing and flowering stage. I_4_ represents two irrigations at jointing and filling stage. I_5_ represents the treatment without irrigation.

Data from 2016 to 2017 have been presented to show the response of LNA and canopy reflectance to different irrigation regimes. Figure 2 shows the change trend of the LNA over growth progress under different experimental treatments in winter wheat. Among all the treatments, the LNA showed in the shape of single-peak over growth progress, reaching the maximum in booting stage. LNA gradually increased with the times of irrigation, resulting in obvious differences among experimental treatments in different growth stages. In addition, LNA under I_5_ treatment was lower than other treatments.

**Figure 3:**
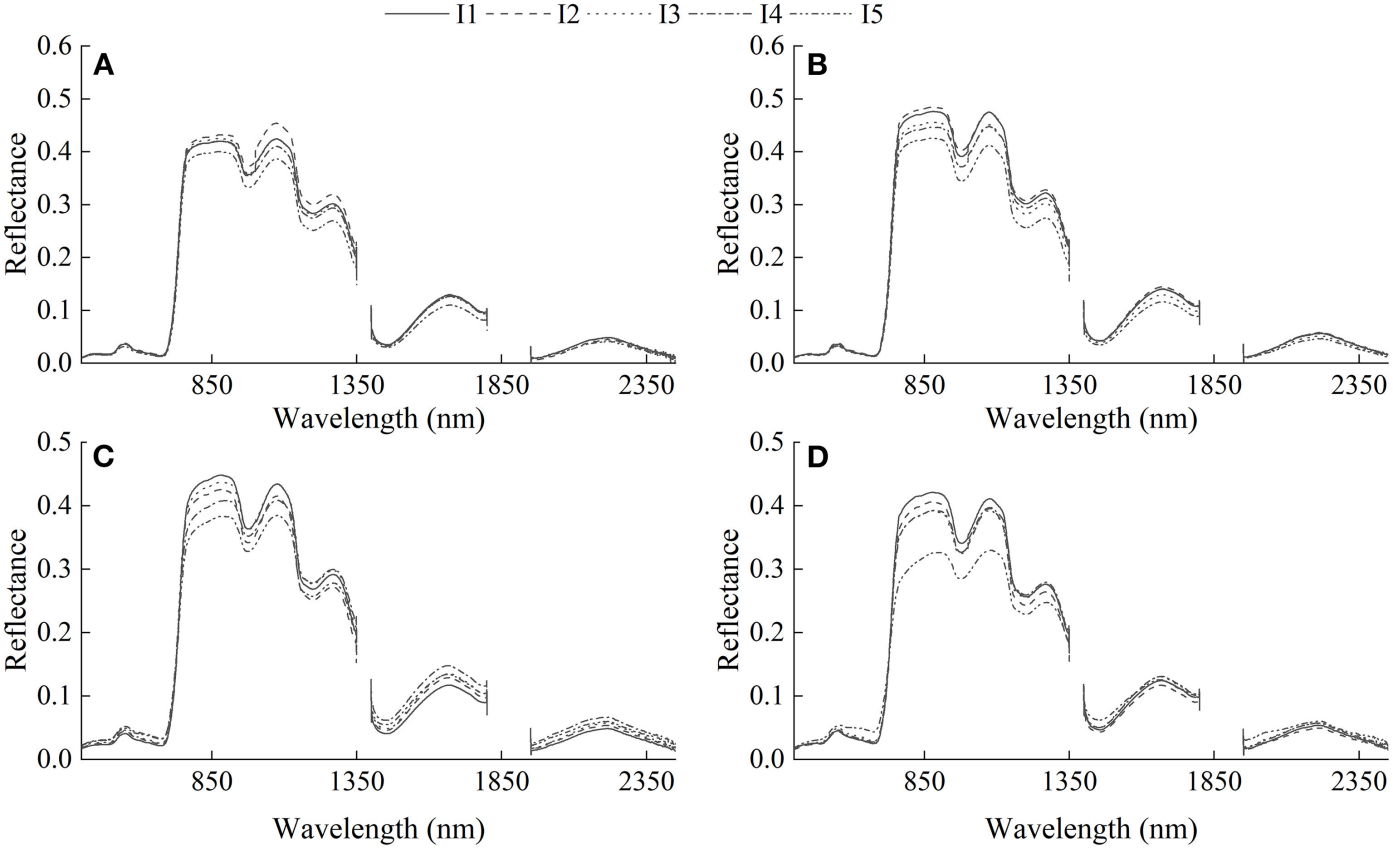
Changes of canopy reflectance with varied irrigation regimes. **(A)** Jointing stage, **(B)** booting stage, **(C)** flowering stage, and **(D)** filling stage. I_1_ represents four irrigations at jointing, booting, flowering, and filling stage. I_2_ represents three irrigations at jointing, booting, and filling stage. I_3_ represents two irrigations at jointing and flowering stage. I_4_ represents two irrigations at jointing and filling stage. I_5_ represents the treatment without irrigation.

The response of canopy reflectance at different growth stages to different irrigation regimes is depicted in Figure 3. It can be seen that canopy reflectance was affected by irrigation application. Canopy reflectance in different spectral regions responded differently to irrigation regimes. Reflectance in the visible region decreased with the times of irrigation and increased in the near-infrared region. The reflectance of the I_5_ treatment was lower than that of other treatments in each growth stage.

### Leaf Nitrogen Accumulation

The statistical analysis of LNA (g/m^2^) is shown in Table 3. The LNA range for the calibration dataset was from 0.99 to 12.56 g/m^2^, and the range for the validation dataset was 1.75–11.44 g/m^2^. Calibration and validation datasets had similar mean value and standard deviation. Furthermore, the *p*-value of K–S test (Lilliefors correction) indicated that the distributions of the calibration and validation datasets were normally distributed with 95% probability. Therefore, the dataset was divided properly and could be used for further analysis.

**Table 3:** Statistics analysis of the leaf nitrogen accumulation (LNA) (g/m^2^) of winter wheat.

**Datasets**	**Number of samples**	**Maximum**	**Minimum**	**Mean**	**Standard deviation**	**p (K-S)**
All observation	241	12.56	0.99	6.576	2.412	0.20
Calibration dataset	160	12.56	0.99	6.577	2.449	0.20
Validation dataset	81	11.44	1.75	6.573	2.351	0.20

### The Relationship Between the Canopy Reflectance and LNA

**Figure 4:**
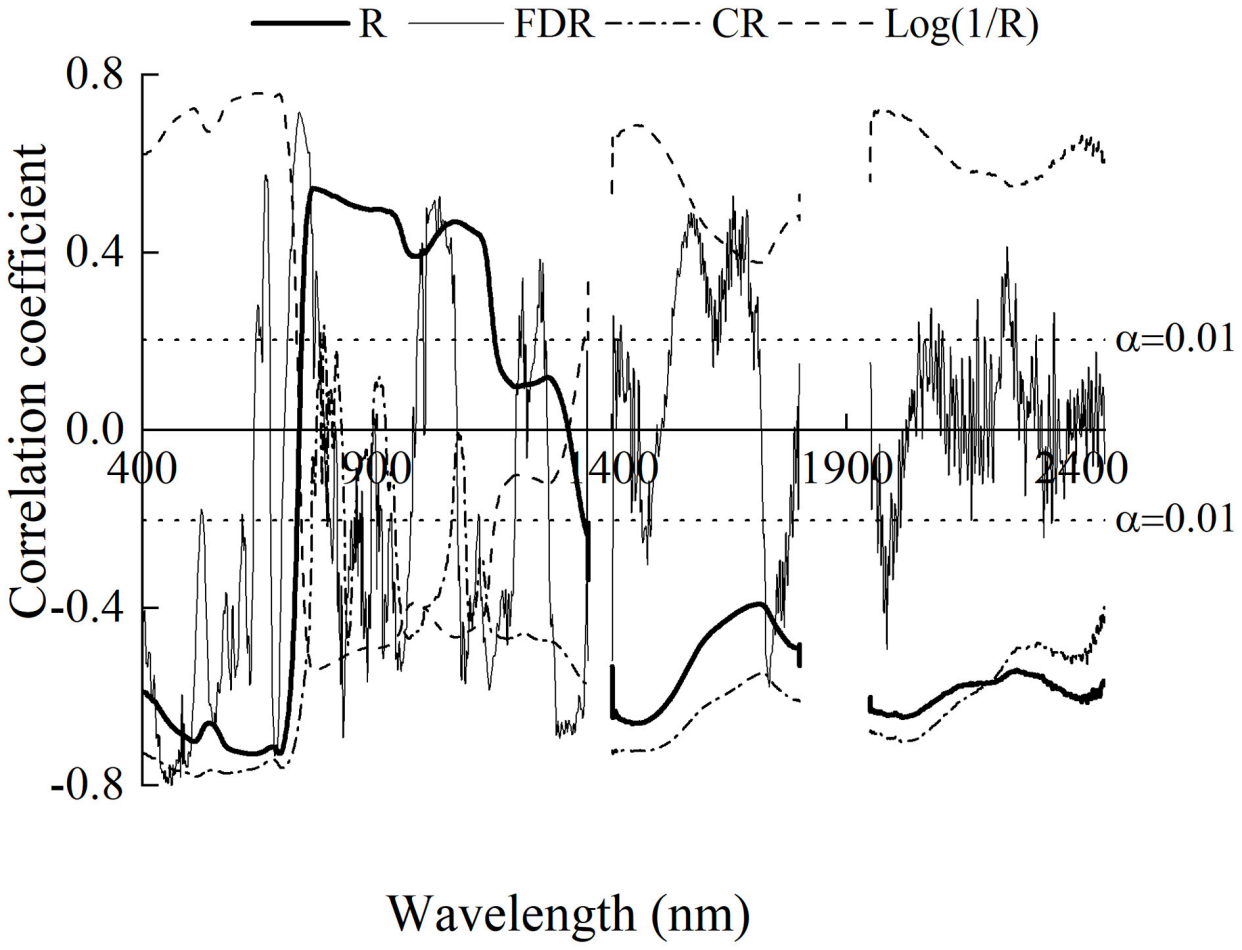
The relationships between leaf nitrogen accumulation and the original reflectance and spectrum with different transformation methods. The dash lines represent the critical value of correlation coefficient at α = 0.01.

Correlations of LNA with OR and three transformed spectral data are shown in Figure 4. LNA showed significant negative correlations between OR in the range of 400–729 and 2,340–2,450 nm, with the strongest correlation at 637 nm (*r* = −0.73). And significant positive correlations were found in 739–1,150 nm, with the largest coefficient at 762 nm (*r* = 0.56). However, the correlation coefficient curve between LNA and Log(1/*R*) was inversely proportional to OR, with closer relationships in 400–700, 1,400–1,500, and 1,950–2,450 nm. It was obvious that CR had improved the correlation in absorption valley ranges of canopy reflectance, and the maximum |r| was 0.78. Different with other transformed spectra, the correlation coefficient between LNA and FDR changed greatly with bands. It also improved the correlation in some ranges, and the maximum |r| was 0.81 at 462 nm. The results indicated that the transformation methods effectively improved the correlation and performed better in estimating LNA.

### Relationships Between the Spectral Indices and LNA

**Figure 5:**
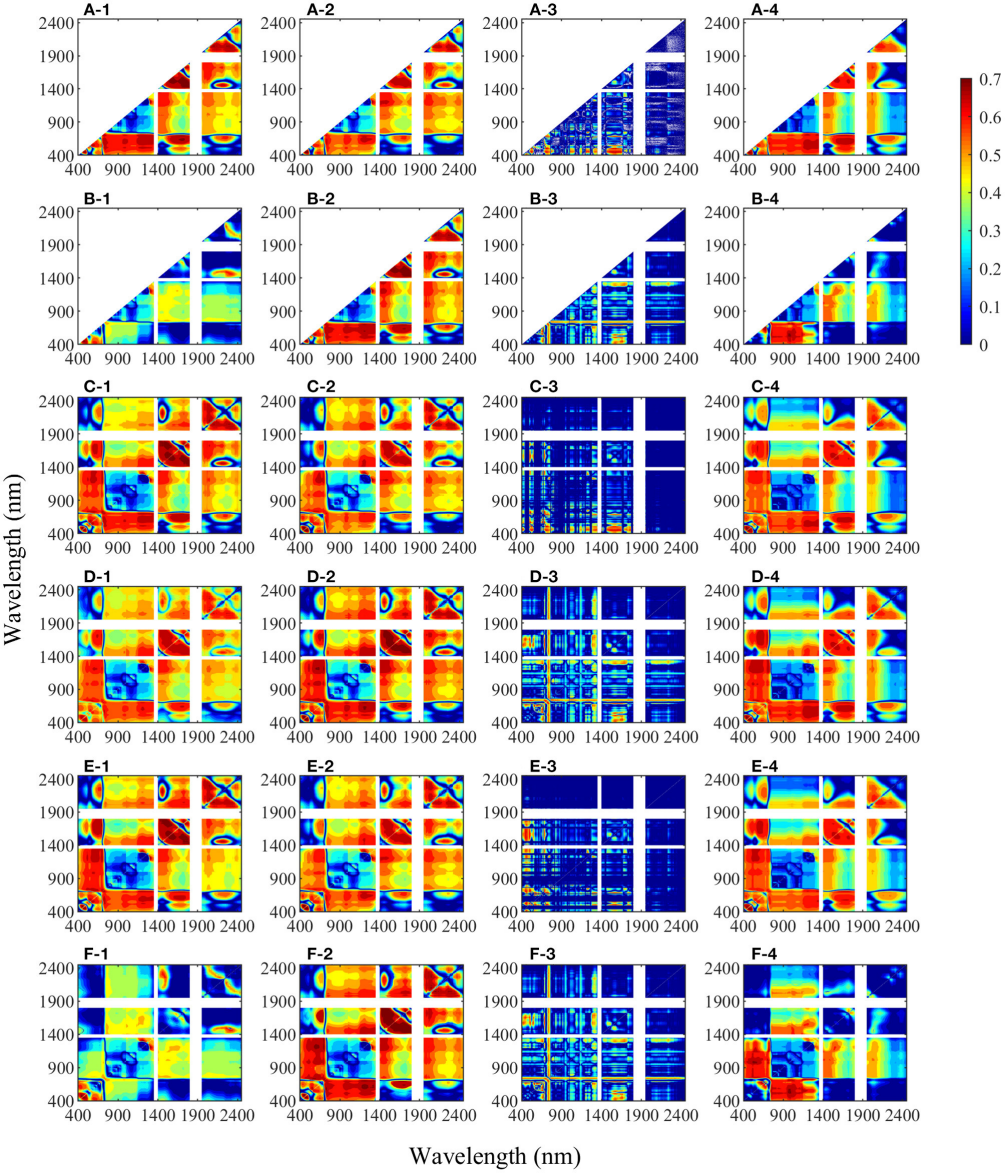
Coefficient of determination (*R*^2^) between leaf nitrogen accumulation and two-band combined spectral indices **(A)** NDSI, **(B)** DSI, **(C)** RSI, **(D)** SASI, **(E)** CSI, and **(F)** MCSI, which were calculated form all possible two bands with different transformation methods in winter wheat (*n* = 160). (1: original reflectance, 2: the logarithm of the reserved reflectance, 3: the first derivative reflectance, and 4: continuum removal spectrum).

Contour maps of the determination coefficients (*R*^2^) for the linear relationship between LNA and two-band spectral indices (i.e., NDSI, DSI, RSI, SASI, CSI, and MCSI, respectively), which were calculated with two random bands in the range of 400–2,450 nm with different transformation methods, are shown in Figure 5. For different spectral indices, the patterns of contour maps of the same transformation method were similar. Except for DSI, the sensitive areas of CR were the largest, and the area (*R*^2^ > 0.6) accounted for about 10–16%, followed by OR with 7.5–12.2% and Log(1/*R*) with 3.5–14.5%. However, only 0.1–1.5% of the area with FDR was >0.6. Compared with other transformations, the sensitive region of FDR spectral indices to LNA was discontinuous, mainly located in the combined areas of visible bands and near-infrared bands. In addition, SASI and MCSI obviously improved the LNA sensitivity of NDSI and CSI, respectively.

**Figure 6:**
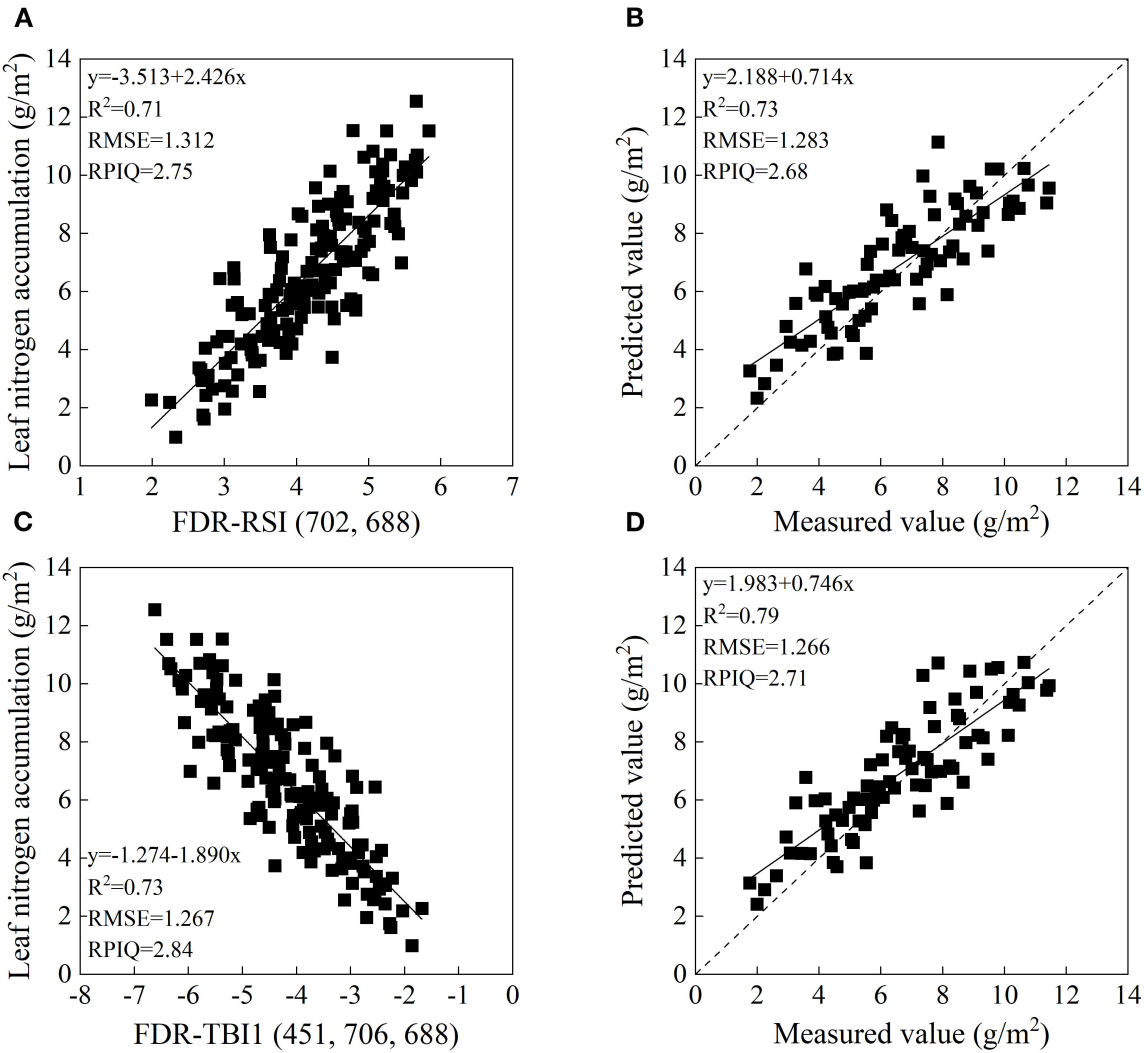
The relationship between leaf nitrogen accumulation (LNA) and FDR-RSI (702, 688) **(A)** and FDR-TBI1 (451, 706, 688) **(C)**. Measured and predicted LNA of the validation dataset based on FDR-RSI (702, 688) **(B)** and FDR-TBI1 (451, 706, 688) **(D)**.

**Figure 7:**
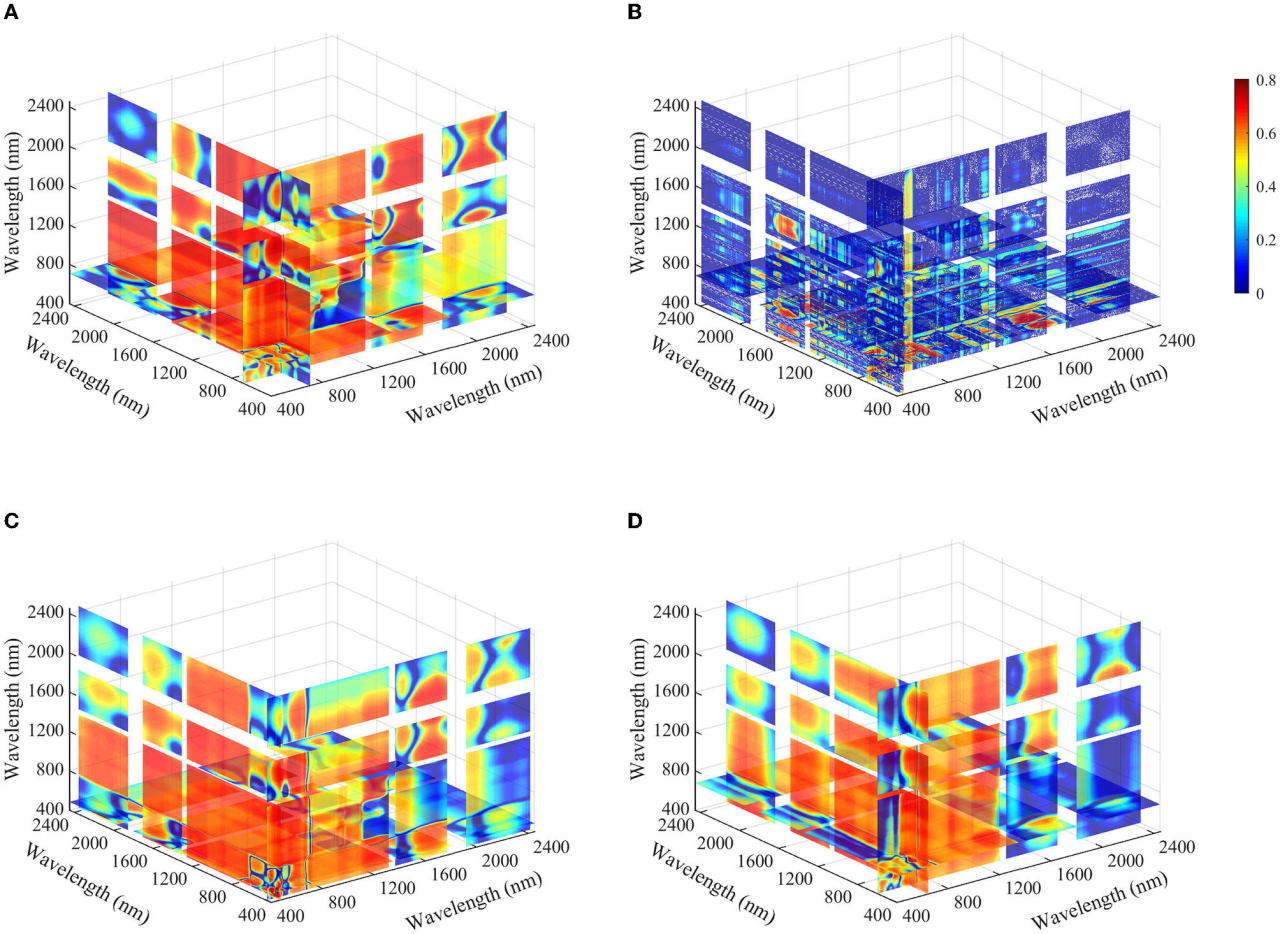
Coefficient of determination (*R*^2^) between leaf nitrogen accumulation and the optimal three-band spectral indices based on different transformation methods **(A)** OR-TBI2 (700, 685, 709), **(B)** FDR-TBI1 (451, 706, 688), **(C)** CR-TBI2 (472, 445, 484), and **(D)** Log(1/R)-TBI2 (643, 598, 676) (*n* = 160).

For each transformed spectra data, the spectral index with the largest *R*^2^ was selected as the optimal spectral index for further analysis. SAVI (450, 458)_(L = 0.10)_, RSI (702, 688), SASI (450, 463)_(L = 0.03)_, and MCSI (465, 460)_(M = −0.6)_ were the optimal two-band indices for OR, FDR, CR, and Log(1/*R*), respectively. As shown in Table 4, models based on the selected indices performed well with *R*^2^ of 0.70–0.73 and 0.73–0.80, and RMSE of 1.288–1.360 and 1.283–1.481 g/m^2^ for calibration and validation, respectively. Relationships between LNA and selected spectral indices were linear, whereas SASI (450, 458)_(L = 0.10)_ generated second-degree polynomial relation. The performance of CR-SASI (450, 463)_(L = 0.03)_ was superior to other two-band spectral indices in model calibration, followed by FDR-RSI (702, 688). However, its RMSE value in validation dataset was increased slightly, indicating inferior accuracy than FDR-RSI (702, 688) and OR-SAVI (450, 458)_(L = 0.10)_. The modeling result showed that FDR-RSI (702, 688) outperformed the other models, with lower RMSE in model calibration and validation (Figures 6A,B). For the TBI, OR-TBI2 (700, 685, 709), FDR-TBI1 (451, 706, 688), CR-TBI2 (472, 445, 484), and Log(1/R)-TBI2 (643, 598, 676) were most closely related to LNA based on different transformed spectra data, with *R*^2^ values of 0.71, 0.73, 0.73, and 0.71, respectively (Figure 7). And the estimating models were all linear model. Consistent with the two-band spectral index, in estimating LNA, the TBI1 based on FDR spectra had superior performance to other transformed spectra data. The model has the *R*^2^ of 0.73 and 0.79, the RMSE of 1.267 and 1.266 g/m^2^, and the RPIQ of 2.84 and 2.71 in calibration and validation datasets, respectively (Figures 6C,D).

**Table 4:** Quantitative models of leaf nitrogen accumulation to selected spectral indices and classical vegetation indices in winter wheat.

**Spectral index**	**Curve shape**	**Calibration dataset**			**Validation dataset**		
		***R*** **^2^**	**RMSE (g/m^**2**^)**	**RPIQ**	***R*** **^2^**	**RMSE (g/m^**2**^)**	**RPIQ**
**Optimal VIs**							
OR-SASI (450, 458) _(L = 0.10)_	Parabolic	0.70	1.360	2.65	0.75	1.375	2.50
FDR-RSI (702, 688)	Linear	0.71	1.312	2.75	0.73	1.283	2.68
CR-SASI (450, 463) _(L = 0.03)_	Linear	0.73	1.288	2.80	0.74	1.404	2.44
Log(1/R)-MCSI (465, 460) _(M = −0.6)_	Linear	0.70	1.349	2.67	0.80	1.481	2.32
OR-TBI2 (700, 685, 709)	Linear	0.71	1.331	2.71	0.72	1.332	2.58
FDR-TBI1(451, 706, 688)	Linear	0.73	1.267	2.84	0.79	1.266	2.71
CR-TBI2(472, 445, 484)	Linear	0.73	1.289	2.79	0.81	1.404	2.44
Log(1/R)-TBI2(643, 598, 676)	Linear	0.71	1.329	2.71	0.78	1.376	2.49
**Classical VIs**							
DCNI	Parabolic	0.42	1.872	1.92	0.46	1.901	1.81
GNDVI	Parabolic	0.61	1.536	2.35	0.59	1.574	2.18
CI_green_	Parabolic	0.58	1.589	2.27	0.58	1.643	2.09
CI_rededge_	Parabolic	0.51	1.729	2.08	0.52	1.748	1.96
EVI2	Parabolic	0.49	1.758	2.05	0.55	1.798	1.91
R810/R660	Logarithm	0.59	1.554	2.32	0.62	1.600	2.15
RI_ldB	Parabolic	0.60	1.536	2.35	0.58	1.581	2.18
MCARI/OSAVI2	Logarithm	0.59	1.556	2.31	0.62	1.601	2.14
OSAVI	Parabolic	0.54	1.666	2.16	0.58	1.691	2.03
NDRE	Parabolic	0.51	1.726	2.09	0.50	1.744	1.97
WRNI	Parabolic	0.68	1.398	2.58	0.68	1.406	2.44
VIopt	Parabolic	0.53	1.699	2.12	0.58	1.730	1.98
NPCI	Linear	0.52	1.704	2.11	0.59	1.649	2.08
(R_924_-R_703_+2*R_423_)/(R_924_+R_703_+2*R_423_)	Parabolic	0.57	1.618	2.23	0.57	1.639	2.09

*OR, FDR, CR, and Log(1/R) represent the original reflectance, the first derivative reflectance, spectra with continuum removal, and the logarithm of reciprocal of spectra, respectively. RSI, SASI, and MCSI represent ratio spectral index, soil adjusted spectral index, and modified chlorophyll spectral index, respectively. TBI represent the three-band spectral index. R^2^, RMSE, and RPIQ represent the coefficient of determination, the root mean square error, and the ratio of performance to interquartile distance, respectively*.

### The Estimation Model of LNA Based on Classical Vegetation Indices

**Figure 8:**
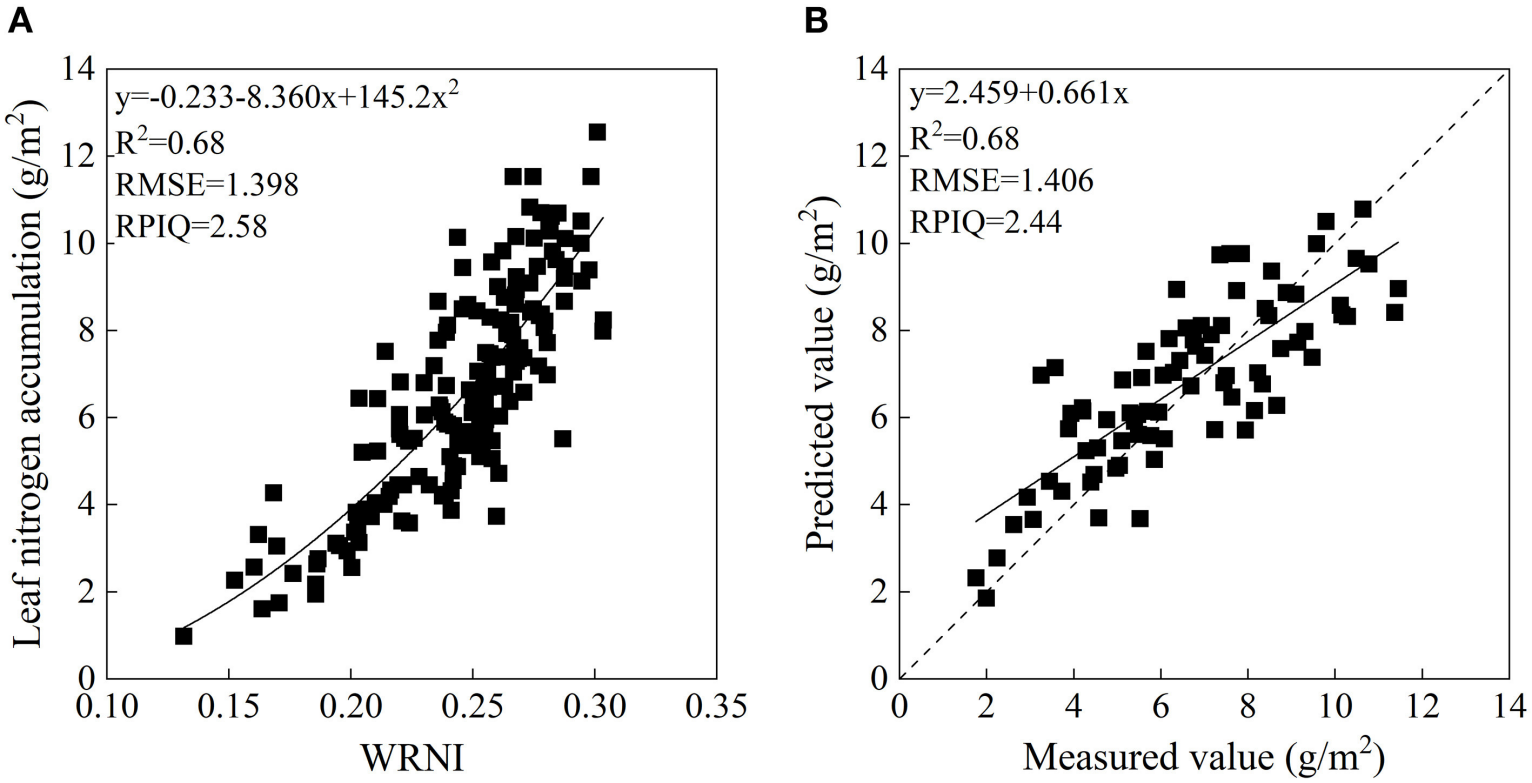
The relationship between leaf nitrogen accumulation (LNA) and WRNI **(A)**. Measured and predicted LNA of the validation dataset based on WRNI **(B)**.

**Figure 9:**
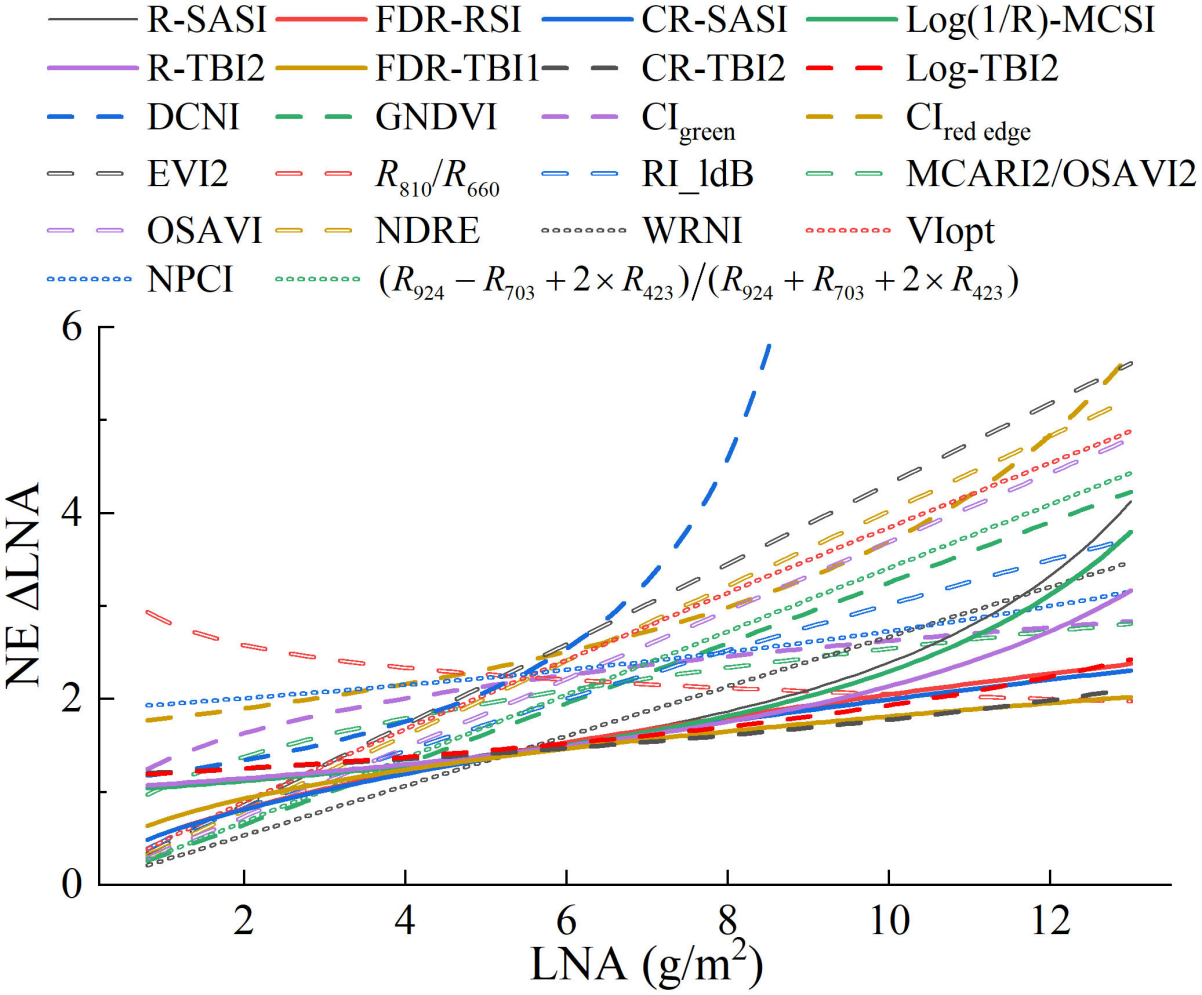
Noise equivalent of leaf nitrogen accumulation (LNA) estimation by different spectral indices.

In order to verify the performance of the optimal spectral index in section the relationship between the canopy reflectance and LNA, relationships between the classical vegetation indices and LNA were studied with calibration and validation datasets. Table 4 demonstrates that the performance of models based on each classical vegetation index is acceptable, with *R*^2^ > 0.50, RMSE < 1.91 g/m^2^, and RPIQ > 1.90 in both model calibration and validation. It demonstrated that these classical vegetation indices were correlated with LNA. And most of the classical indices had a non-linear relationship with LNA. The top three indices for *R*^2^ of calibration model were water resistance N index (WRNI), GNDVI, and RI_ldB, all of which were >0.60. Among the classical vegetation indices, the performance established on the WRNI exhibited the highest accuracy in estimating LNA, with the highest *R*^2^ and RPIQ and the lowest RMSE in calibration and validation (Figure 8). Furthermore, the NE was compared to evaluate the sensitivity of optimal spectral indices to LNA. As shown in Figure 9, the NE value increased along with the LNA, except for R810/R660. The optimal spectral indices selected in section relationships between the spectral indices and LNA had relatively stable NE value, and they had higher sensitivity than other classical vegetation indices when the LNA was above 5.0 g/m^2^.

### Effects of Irrigation Regimes on the Spectral Index Performance in Estimating LNA

In order to study the performance of spectral indices under different irrigation regimes, three well-performing spectral indices (FDR-RSI (702, 688), FDR-TBI1 (451, 706, 688), and WRNI) were selected and compared. As shown in Table 5, the correlations between spectral indices and LNA under different irrigation regimes are different. Spectral indices were most closely related to the LNA under the I_5_ treatment, with *R*^2^ ranging from 0.81 to 0.85. In term of different irrigation regimes, FDR-RSI (702, 688) had the largest *R*^2^ at the I_3_ treatment, whereas the *R*^2^ value of FDR-TBI1 (451, 706, 688) was the largest under other treatments. As a whole, the optimized TBI [FDR-TBI1 (451, 706, 688)] was the best spectral index for LNA estimation under different irrigation regimes.

**Table 5:** Relationships between spectral indices and leaf nitrogen accumulation under different irrigation regimes.

**Treatments**	**FDR-RSI (702, 688)**			**FDR-TBI1(451, 706, 688)**			**WRNI**		
	***R*** **^2^**	**RMSE (g/m^**2**^)**	**RPIQ**	***R*** **^2^**	**RMSE (g/m^**2**^)**	**RPIQ**	***R*** **^2^**	**RMSE (g/m^**2**^)**	**RPIQ**
I1	0.64	1.353	2.61	0.65	1.328	2.66	0.50	1.582	2.23
I2	0.64	1.453	2.53	0.66	1.374	2.64	0.54	1.594	2.28
I3	0.71	1.226	2.56	0.70	1.245	2.52	0.61	1.416	2.21
I4	0.67	1.255	2.33	0.69	1.213	2.41	0.67	1.250	2.34
I5	0.81	1.122	2.36	0.85	1.054	3.58	0.82	1.130	3.34

## Discussion

Canopy reflectance are various with different irrigation regimes (Figure 3). The growth of winter wheat was influenced by irrigation, resulting in the difference of canopy reflectance. With the decrease of irrigation times, the reflectance in the visible region tended to increase, whereas the reflectance in the near-infrared region gradually decreased. These results agree with the findings of Feng et al. (2013), who reported the reflectance is greater in the visible region and lower in the near-infrared region under drought stress. It may be related to that water stress caused a decrease in leaf area, water status, and chlorophyll content (Jaleel et al., 2009).

Canopy reflectance is the comprehensive information in the observation field, including the target object and the underlying surface. Therefore, it contains the information of interest and the information of interference. The application of proper spectral preprocessing can generate good prediction accuracy (Li L. et al., 2016; Li et al., 2020). In this study, the spectra processed with three transformation methods had improved the correlation coefficient with LNA (Figure 4), and the transformed-based spectral index had better performance in LNA estimation (Table 4). Specifically, CR improved the correlation coefficients in the visible region predominantly influenced by chlorophyll pigments. Haboudane et al. (2002) reported that the visible region is closely related to leaf nitrogen status. The result can be explained by that CR could highlight and identify more absorption characters of the target traits (Huang et al., 2004). Based on this region, the two- and three-band CR-based spectral indices performed well in the model calibration. The result was similar to that of the study of Li D. et al. (2016), which reported that the CR-based spectral indices had better performance in estimating leaf nitrogen content of litchi. As for Log(1/*R*), it simply but roughly linearized the absorption effect, without considering the multiple scattering (Dawson et al., 1999). Therefore, it has shown the similar contour maps to OR (Figure 5), and the optimal spectral indices have slightly increased relationship to LNA as shown in Table 4. Although the FDR has the smallest sensitive regions as shown in Figure 5, the FDR-based optimal index had good and stable performance. It may be because that the FDR could effectively reduce the effect of soil background (Meng et al., 2020) and emphasize the week but meaningful peaks (Shibayama et al., 1993). For example, FDR significantly improved the correlation coefficients in the red-edge region, which was founded to be sensitive to nitrogen status (Li et al., 2014). Using the FDR in this region, the optimized two- and three-band spectral indices were superior to other indices. Moreover, the transformed-based spectral indices had linear relationships with LNA, having lower and more stable NE values than spectral indices based on OR (Figure 9). It indicated that transformed spectra may had a positive effect on reducing the saturation phenomenon. This further illustrated the advantages of the spectral index calculated with transformed spectra from another aspect.

To mine more important band combinations related to LNA, the entire range of spectral data (400–2,450 nm, except for ranges disturbed by atmosphere and machine noise) was used to construct the two- and three-band spectral indices. The optimal TBI [FDR-TBI1 (451, 706, 688)] presented better performance than the optimal two-band spectral [FDR-RSI (702, 688)]. The result was identical with previous studies reported by Shi et al. (2016) and Wang et al. (2019), in which three-band vegetation index can more accurately estimate the target variable. It is noted that, in this study, optimized two-band spectral indices contained one spectral region, whereas the TBIs had a wider band distribution and covered more than one spectral region. Therefore, one possible reason for the better performance of the TBI is that three bands in blue and red-edge regions carried more information of LNA. Another reason may be concerned with that bands used in the spectral index were more informative and effective. With the highest correlation with LNA and the best model performance, FDR-TBI1 (451, 706, 688) was selected as the optimal spectral index for estimating LNA of winter wheat under different irrigation regimes. The result was consist with studies of Hansen and Schjoerring (2003) and Peñuelas et al. (1994), which proved that the combination of blue bands and red bands was effective in nitrogen status evaluation. The band 451 nm was located in the absorption of chlorophyll and carotenoid. Since nitrogen is one of the components of protein, which had a positive function on photosynthesis, the sensitive bands of chlorophyll were correlated with plant nitrogen content (Filella et al., 1995; Pettersson and Eckersten, 2007; Clevers and Gitelson, 2013). Two bands (688 and 706 nm) were located in the red-edge region (680–760 nm), which has been found to be effective in nitrogen status estimation (Li et al., 2014). Among classical vegetation indices, better model performance and lower NE were obtained with the vegetation index constructed with red-edge bands (e.g., RI_ldB and WRNI). Similarly, based on the OR and Log(1/*R*), the TBIs with red-edge band were superior to the corresponding two-band spectral indices. Such a result can be explained by the fact that the red-edge region was much sensitive to LNA and may maintain higher sensitivity of spectral index when LNA was high (Magney et al., 2017; Hasituya et al., 2020).

The effect of water status should be considered into the plant nitrogen estimation (Feng et al., 2016). Feng et al. (2016) developed new index (WRNI) by combining an optimized NDRE and FWBI to mitigate the impact of water content on nitrogen monitoring. Our experiments were carried out under different irrigation regimes, so the effect would exist. As it would be expected, WRNI had the best performance in model calibration and validation among classical vegetation indices in section relationships between the spectral indices and LNA. The RMSE of the new developed index FDR-TBI1 (451, 706, 688) was 9% and 10% lower RMSE than that of WRNI in model calibration and validation, respectively. It may be due to the fact that WRNI was developed for leaf nitrogen content. Because of the different agronomic compositions, there had difference in sensitive spectral regions (Chu et al., 2014). In addition, a previous study had reported that 688 nm can also be used to monitor water content (Sun et al., 2021). The band 688 nm appeared in the denominator of the optimal spectral index, which may made it to dynamically reflect the changes of LNA in different water status. In practical applications, the new spectral index requires fewer bands, which is more conducive to the development of low-cost instrument.

The relationships between LNA and spectral indices were different under different irrigation regimes, with the best correlation under the I_5_ treatment (Table 5). A similar phenomenon was founded in the study performed by El-Hendawy et al. (2019), who showed that spectral indices had a stronger correlation with aboveground dry biomass and grain yield under the limited water irrigation than under the full irrigation. Presumably, it is because of the difference in irrigation. The water status of plant changed with irrigation regimes, and the accuracy of nitrogen status estimation was affected by leaf water content. In the study of Feng et al. (2016), the NDRE had higher performance of leaf nitrogen content at lower leaf water content. Similar result was shown in this study, with spectral index tending to be more correlated with LNA when leaf water content was lower (Supplementary Table 1). During the growth period of winter wheat, the water status was lower than other treatments under the I_5_ treatment (Sun et al., 2021), which contributed to a closer relationship between spectral indices and LNA.

Despite using the datasets, including three cultivars, 2 years, and multiple growth stages, it should be noted that the optimized spectral index in this study was developed based on winter wheat in one cultivated ecological environment. Thus, the reliability and adaptability of the spectral index need to be investigated in more crop types and in more ecological regions. In addition, the bands used in the optimized spectral index overlapped with existing hyperspectral satellites (e.g., HJ-1A HSI) and airborne sensors (e.g., RedEdge-MX Dual and Cubert UHD185). Hyperspectral satellite technology and the UAV technology had rapidly developed, and the bands of imaging spectrometer would be narrower with higher spatial resolution. Therefore, the optimized spectral index had great potential in the large-area LNA estimation with satellite and UAV platforms.

## Conclusion

In this study, the results demonstrated that the spectral transformation method can effectively improve the relationship between spectral data and LNA. Compared with OR, the transformation-based spectral indices had more stable and higher sensitivity and performed better in estimating LNA, indicating that it is advantageous to apply transformed spectral data to the construction of spectral index. FDR was proved to be the best transformation method for spectral index construction. Compared to classical vegetation indices and optimized spectral indices, FDR-TBI1 (451, 706, 688) which had a linear relationship with LNA, had the best and stable performance in estimating LNA. Simultaneous optimization of spectral data and bands provides us an effective way of constructing spectral index. The optimized spectral index FDR-TBI1 (451, 706, 688) can provide accurate nitrogen prediction for winter wheat under different irrigation regimes. The results can provide technical supports for large area nitrogen monitoring of winter wheat.

## Data Availability Statement

The original contributions presented in the study are included in the article/Supplementary Material, further inquiries can be directed to the corresponding authors.

## Author Contributions

HS: conceptualization, methodology, software, validation, formal analysis, writing—original draft, and visualization. MF: resources, investigation, and funding acquisition. WY: investigation, supervision, project administration, and funding acquisition. RB: project administration and writing—review and editing. JS: methodology and writing—review and editing. CZ: investigation, formal analysis, and data curation. LX: methodology and validation. CW and MK: validation and data curation. All authors contributed to the article and approved the submitted version.

## Funding

The authors are grateful to the support by the National Natural Science Foundation of China (31871571 and 31371572), the Key Technologies R&D Program of Shanxi province (201903D211002-01, 05), the Basic Research Program of Shanxi province (20210302123411 and 20210302124236), the Doctoral Research Project of Shanxi Agricultural University (2021BQ99 and 2020BQ32), the Applied Basic Research Project of Shanxi province, China (201801D221299), and the Outstanding Doctor Funding Award of Shanxi province (SXYBKY2018040).

## Conflict of Interest

The authors declare that the research was conducted in the absence of any commercial or financial relationships that could be construed as a potential conflict of interest.

## Publisher's Note

All claims expressed in this article are solely those of the authors and do not necessarily represent those of their affiliated organizations, or those of the publisher, the editors and the reviewers. Any product that may be evaluated in this article, or claim that may be made by its manufacturer, is not guaranteed or endorsed by the publisher.

## Abbreviations

CR: continuum removal
CSI: chlorophyll spectral index
DSI: difference spectral index
FDR: the first derivative reflectance
LNA: leaf nitrogen accumulation
MCSI: modified chlorophyll spectral index
NDSI: normalized difference spectral index
*R* ^2^: coefficient of determination
RMSE: root mean square error
RPIQ: the ratio of performance to interquartile distance
RSI: ratio spectral index
SASI: soil-adjusted spectral index
CI: chlorophyll index
MCI: modified chlorophyll index
OR: original reflectance
WRNI: water resistance N index
NE: noise equivalent
TBI: three-band spectral index
NDRE: normalized difference red-edge index.
