# Supplementary material for: Monitoring Leaf Nitrogen Accumulation With Optimized Spectral Index in Winter Wheat Under Different Irrigation Regimes
Source: Front Plant Sci. 2022 Jun 17;13:913240. doi: 10.3389/fpls.2022.913240 (PMC9247552; doi:10.3389/fpls.2022.913240)
Supplement: Supplementary file 1 [file Table_1.docx]

Table S1 | Relationships between spectral indices and leaf nitrogen accumulation with different leaf water content (%)

| Categories | FDR-RSI (702, 688) | | FDR-TBI1(451, 706, 688) | | WRNI | |
| --- | --- | --- | --- | --- | --- | --- |
|  | R^2^ | RMSE (g/m^2^) | R^2^ | RMSE (g/m^2^) | R^2^ | RMSE (g/m^2^) |
| >80 | 0.12 | 1.358 | 0.20 | 1.295 | 0.07 | 1.392 |
| 70-80 | 0.55 | 1.234 | 0.56 | 1.224 | 0.51 | 1.291 |
| <70 | 0.30 | 1.123 | 0.36 | 1.077 | 0.48 | 0.973 |
